# A machine learning model to predict optimal antibiotic use in hospital medicine patients

**DOI:** 10.1017/ash.2025.10142

**Published:** 2025-09-29

**Authors:** Nathan Radakovich, Priya Prasad, Daniel Escobar, Ripal Jariwala, Allison Bond, Sandra Oreper, Junghwan Lee, Xiao Hu, Sarah Doernberg

**Affiliations:** 1 Department of Medicine, University of California, San Francisco, CA, USA; 2 Division of Infectious Diseases, Department of Medicine, University of California, San Francisco, CA, USA; 3 Georgia Institute of Technology, Atlanta, GA, USA; 4 Emory University, Center for Data Science, Atlanta, GA, USA

## Abstract

**Objective:**

Unnecessary and suboptimal antibiotic use causes adverse outcomes at both the level of individuals and health systems. Prospective audit and feedback, a core aspect of antibiotic stewardship program (ASP) efforts, reduces such use, but is inefficient in the absence of a pre-screening process. To address this, we used a machine learning approach to stratify antibiotic orders based on the likelihood of benefiting from ASP review, and to identify the factors most influential in the model’s predictions.

**Design:**

Machine learning model developed using expert-labeled point-prevalence data.

**Setting:**

Single-center adult academic hospital.

**Participants:**

Hospitalized patients 18 years or older on internal medicine services between May 2021 and August 2022.

**Methods:**

Infectious disease experts assessed antibiotic orders for necessity and optimal use to create labels which were used as ground truth to train a machine learning model that uses automatically queried data from the electronic health record including vital signs, laboratory values, microbiological data, and medication administration records.

**Results:**

Our model achieved an area under the receiver operating characteristic curve of 0.89 for antibiotic necessity and 0.80 for optimal use. Model predictions were driven largely by markers of clinical instability, inflammation, and infection. Simpler clinical indices of infection had no predictive ability.

**Conclusions:**

We describe a model that predicts antibiotic necessity and optimal use using routinely available data that can be automatically aggregated from medical records. This makes it a promising option for early identification and intervention on orders most likely to benefit from ASP review.

## Introduction

As many as 50% of hospitalized patients receive antibiotics during the course of their hospitalization.^1^ Despite antibiotics’ life-saving role in treating bacterial infections, approximately one fifth of hospitalized patients who receive them experience adverse effects, including hypersensitivity reactions, direct toxicity, and *Clostridioides difficile* infection.^2,3^ On a systems level, broad-spectrum antibiotic use drives increased rates of multi-drug resistance, contributing to worldwide morbidity and mortality.^4^ In hospital settings, where an estimated 30% of antibiotic use is unnecessary, some of this harm may be avoidable.^5^ Antibiotic stewardship programs (ASPs) seek to balance antibiotics’ utility with the harms stemming from unnecessary or suboptimal use through restriction of certain antibiotics, prospective audit and feedback (PAF), and co-implementation with infection control measures.^6^ ASP interventions have been shown to decrease rates of both multidrug-resistant organisms and incidence of *C. difficile* infection, and have an important role to play in reducing the adverse consequences of suboptimal antibiotic use.^7^

Prospective audit and feedback (PAF), a backbone of ASP efforts, optimizes antibiotic use via expert review of medical records for appropriateness and via proactive outreach to prescribing teams.^8^ This includes identifying cases where antibiotics are not indicated, or where modifying them might reduce toxicity or improve efficacy. PAF reduces the use of broad-spectrum antibiotic use and might reduce rates of antibiotic resistance without negatively affecting outcomes.^2,9,10^ Despite PAF’s benefits, its labor-intensive nature limits higher-volume implementation.^11^ An automated method to stratify orders based on their need for review might refocus ASP audits toward orders most likely to benefit from interventions.

To this end, we developed a machine learning model to flag suboptimal or non-indicated antibiotic orders for ASP audit and feedback. The model uses data that can be automatically queried from the electronic health record (EHR), and makes clinician-interpretable predictions about indications and optimal antibiotic use.

## Methods

### Study population

Data collection and analysis for this study were approved by the University of California, San Francisco (UCSF) internal review board. Our study cohort included adult patients receiving at least one antibiotic who were treated on the hospital medicine service between May 2021 and August 2022 at UCSF Parnassus Medical Center, an adult tertiary academic hospital. Separate medical services (intensive care, cardiology and malignant hematology) were excluded, as were surgical services. Three infectious diseases experts (two faculty physicians and one infectious diseases specialty pharmacist) completed quarterly periodic point prevalence surveys of all patients on a given medicine team receiving antibiotics on the day of the survey. A single clinician assessed orders for (1) whether antibiotics were indicated and (2) whether the antibiotic selected was optimal. To ensure uniformity, all reviews were performed using the National Antimicrobial Prescribing Survey (NAPS) tool, a standardized, clinically validated questionnaire.^12^ National Antimicrobial Prescribing Survey (NAPS) assesses antimicrobial agent indication and optimal use including dose, route of administration, clinical syndrome, patient allergies/comorbidities, and microbiological data, if applicable (a complete version of the tool is in the supplemental appendix).

Reviewers at our institution assessed orders based on patient notes, vital signs, laboratory values, imaging, medicine administration record (MAR), and microbiological data. Data were collected and processed at the level of individual antibiotic orders. Two concurrent orders for the same patient were considered separately—due to the intermittent point prevalence design of the reviews, repeat orders for the same patient were assessed at intervals of 3 months or greater, and were therefore considered independently of one another. Patients receiving infectious disease consultation were excluded from review.

### Data extraction, labeling, and descriptive statistics

Data were extracted from the medical record via a reporting database, Clarity, attached to our institution’s EHR (Epic Systems Corporation, Verona, Wisconsin, United States). To match the model’s intended use case (ie, automated screening of existing/new antibiotic orders), data were censored after 11:59 PM on the day prior to review. Subsequent data processing and machine learning model development were done using the Python programing language (Python 3.9.2, Python Software Foundation, Wilmington, Delaware, United States); libraries used are detailed in the supplement. Student’s *T*-test or the Kruskal–Wallis test were used to compare continuous variables, the chi-squared test or Pearson’s exact test for categorical ones.

### Model generation

We selected the initial pool of training features based on their relevance to infection. Candidate features included: markers of hemodynamic and respiratory status (eg, vital signs, pulse oximetry); inflammatory markers (white blood cell count (WBC), C-reactive protein (CRP), eg,); and markers of end organ function (creatinine, bilirubin, eg,). A complete medication administration record (MAR), including antibiotics, immunosuppressants, and microbiological data were also included. medication administration record (MAR) data contained date and time of administration for antibiotics, allowing duration of treatment to be determined for each. Microbiological data included species, presence of extended-spectrum beta lactamase producing enterobacterales (ESBL; designated by presence of ceftriaxone resistance), identification of methicillin-resistant *Staphylococcus aureus* isolates (MRSA; designated by either phenotypic testing or rapid molecular diagnostics), and for negative cultures, the quantity of time with no growth detected.

We selected a machine learning approach to develop our model based on machine learning models’ ability to capture hierarchical, nonlinear relationships without explicit *a priori* instruction about how to relate variables to one another.^13^ The model chosen, XGBoost, makes predictions by iteratively creating decision trees to generate and refine predictions.^14^ The model used a 10-fold cross-validation scheme with non-repeating partitions of 90% train and 10% test data to prevent overfitting. The performance metrics reported herein are aggregated from the 10 different partitions of test data, which were not used to directly train models.

Area under the receiver operating characteristic curve (AUROC) was used to adjudicate predictive ability—AUROC measures the ability to distinguish between different types of objects, with an AUROC of 1.0 representing a perfect predictor and an AUROC of 0.5 representing a predictor that performs no better than random chance (an AUROC 0.5–0.6 is generally considered poor, 0.6–0.7 weak, 0.7–0.8 fair, 0.8–0.9 good, and 0.9–1.0 excellent, although these are not formal definitions and performance needs to be considered in context). SHAP (SHapley Additive exPlanations), an approach to machine learning model interpretation that uses game theory to identify variables’ relative influence on model predictions, was used both to iteratively select variables during model development and to interpret the final model’s predictions.

After model generation and evaluation we assessed the performance of the systemic inflammatory response syndrome (SIRS) criteria, a commonly used diagnostic tool for infections in hospitalized patients, to query whether our model’s performance exceeded that of simpler clinical decision tools; the quick sepsis-related organ failure assessment score was not used due to the quantity of missing data for Glasgow Coma Scale measurements.^15–17^

Study design, model generation and data reporting were executed in alignment with the TRIPOD guidelines for clinical prediction models.^18^ The supplemental appendix describes additional details about variable selection, feature engineering and model development.

## Results

425 antibiotic orders were reviewed and annotated between May 2021 and August 2022. 176 (41%) of patients were female, the average age was 64 years (interquartile range (IQR): 53–74), and the median duration of hospitalization prior to review was 11 days (IQR: 7–15). Of orders reviewed, 317 (75%) and 236 (56%) were assessed as indicated and optimal, respectively. Demographic, vital sign, laboratory and antibiotic data are summarized in Table 1. No statistically significant differences were seen between antibiotics received, vital sign values, laboratory values, or demographic characteristics between patients who met the “indicated” versus “optimal” criteria (unadjusted *P* > 0.05 for all comparisons).

**Table 1.** Cohort characteristics

	Optimal		Indicated	
	Yes	No	Yes	No
Female	96 (22)	80 (18)	136 (32)	37 (8)
BSHO	100 (23)	71 (16)	135 (32)	35 (8)
BSCA	159 (37)	141 (33)	218 (52)	74 (17)
Gram positive	172 (40)	142 (33)	239 (57)	65 (15)
MRSA	172 (40)	142 (33)	239 (57)	65 (15)
ESBL	93 (21)	59 (13)	124 (29)	26 (6)
Pseudomonas	117 (27)	96 (22)	160 (38)	50 (12)
Positive blood culture	58 (13)	34 (8)	75 (18)	11 (2)
Positive urine culture	74 (17)	72 (16)	112 (26)	30 (7)
Age	65 (53–74)	64 (54–75)	64 (55–75)	63 (52–73)
Days of antibiotics	1 (1–5)	1 (1–4)	1 (1–4)	1 (0–3)
ASI at review	9 (5–13)	10 (6–14)	9 (5–13)	9 (5–13)
Median SBP, 24h	121 (110–135)	119 (106–132)	121 (107–133)	120 (108–137)
Median HR, 24h	84 (71–96)	89 (77–99)	84 (73–98)	90 (80–99)
Median RR, 24h	18 (16–18)	18 (16–18)	18 (16–18)	18 (16–18)
Median temperature, 24h	98 (97–98)	98 (97–98)	98 (97–98)	98 (97–98)
Median WBC, 24h	8 (6–13)	9 (7–14)	8 (6–13)	9 (6–15)
Median platelet count, 24h	221 (143–338)	240 (156–324)	228 (142–338)	231 (158–290)
Median lactate, 24h	1 (1–2)	1 (1–1)	1 (1–1)	1 (1–2)

Parentheses show column percentages for categorical variables and interquartile ranges for continuous ones. BSHO: broad-spectrum hospital onset, HSCA: broad-spectrum community acquired, MRSA: methicillin-resistant *Staphylococcus aureus*, ESBL: extended-spectrum beta lactamase, ASI: antibiotic spectrum index, SBP: systolic blood pressure, HR: heart rate (beats per minute), RR: respiratory rate (breaths per minute), WBC (1 000 cells per microliter).

**Figure 1.**
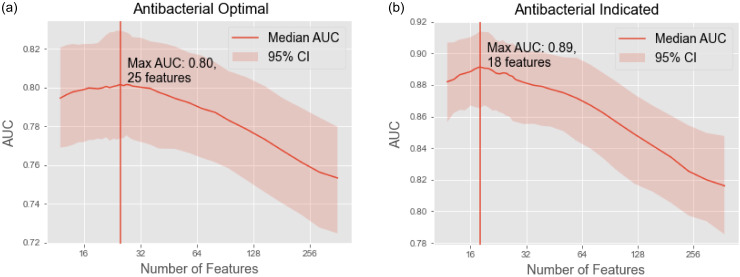
Feature selection and optimization. 1a and 1b show AUROC trend as non-informative features are iteratively removed; trend lines represent a rolling average of AUROC, with 95% confidence interval (CI) depicted by shading.

Stepwise feature selection identified optimum performance at 18 features for both the indicated and optimal criteria, yielding respective AUROCs (95% confidence interval) of 0.89 (0.87–0.91) and 0.80 (0.77–0.83) (Figure 1). To explore which types of clinical data were most informative for making predictions, we also trained separate models using: vital signs only; laboratory values only; and microbiology/antibiotic administration data only (these were paired to assess how well the model could learn to identify concordance between culture data and spectra of coverage).

For the final models (ie, those using all available data that had undergone feature selection to optimize performance), we also assessed performance on different subsets of antibiotics as defined by the National Healthcare Standardized Antimicrobial Administration Ratios antimicrobial categories: broad-spectrum hospital onset; broad-spectrum community onset; MRSA; ESBL and *Pseudomonas aeruginosa*.^19^ In brief, model performance was highest using all available data, and lower when trained individually on vitals, laboratory data, and microbiological/MAR data, in descending order of performance. This held true for both predicting whether antimicrobials were indicated and for whether the optimal antimicrobial was being used. Model performance across spectra of coverage appeared similar. For ease of readability, model performance is detailed in Table 2. Systemic inflammatory response syndrome (SIRS) criteria distributions are shown in Figures S3a and S3b.

**Table 2.** Model performance

	Indicated		Optimal	
Training data	AUROC	95% CI	AUROC	95% CI
All data	0.89	(0.87–0.91)	0.8	(0.77–0.83)
Vital signs	0.87	(0.70–0.98)	0.77	(0.58–0.91)
Laboratory data	0.76	(0.57–0.93)	0.76	(0.540.90)
Microbiology/antibiotics	0.74	(0.45–0.98)	0.66	(0.48–0.82)
SIRS criteria	0.42	(0.37–0.49)	0.42	(0.36–0.47)
Spectrum of coverage	AUROC	95% CI	AUROC	95% CI
BSHO	0.86	(0.78–0.92)	0.77	(0.70–0.86)
BSCA	0.87	(0.80–0.90)	0.81	(0.75-–0.85)
MRSA	0.87	(0.82–0.92)	0.77	(0.71–0.84)
ESBL	0.89	(0.78–0.97)	0.73	(0.64–0.82)
PsA	0.82	(0.73–0.88)	0.78	(0.70–0.83)

Area under the receiver operating characteristic curve (AUROC) with 95% confidence intervals (CI) are listed, with performance stratified by the profile of data used to train the model (all available data vs subsets) as well as model performance by spectrum of coverage (broad-spectrum hospital onset (BSHO), broad-spectrum community onset (BSCO), methicillin-resistant *Staphylococcus aureus* (MRSA), extended-spectrum beta-lactamase producing organisms (ESBL) and *Pseudomonas aeruginosa* (PsA). The systemic inflammatory response (SIRS) criteria are listed as a comparator.

**Figure 2.**
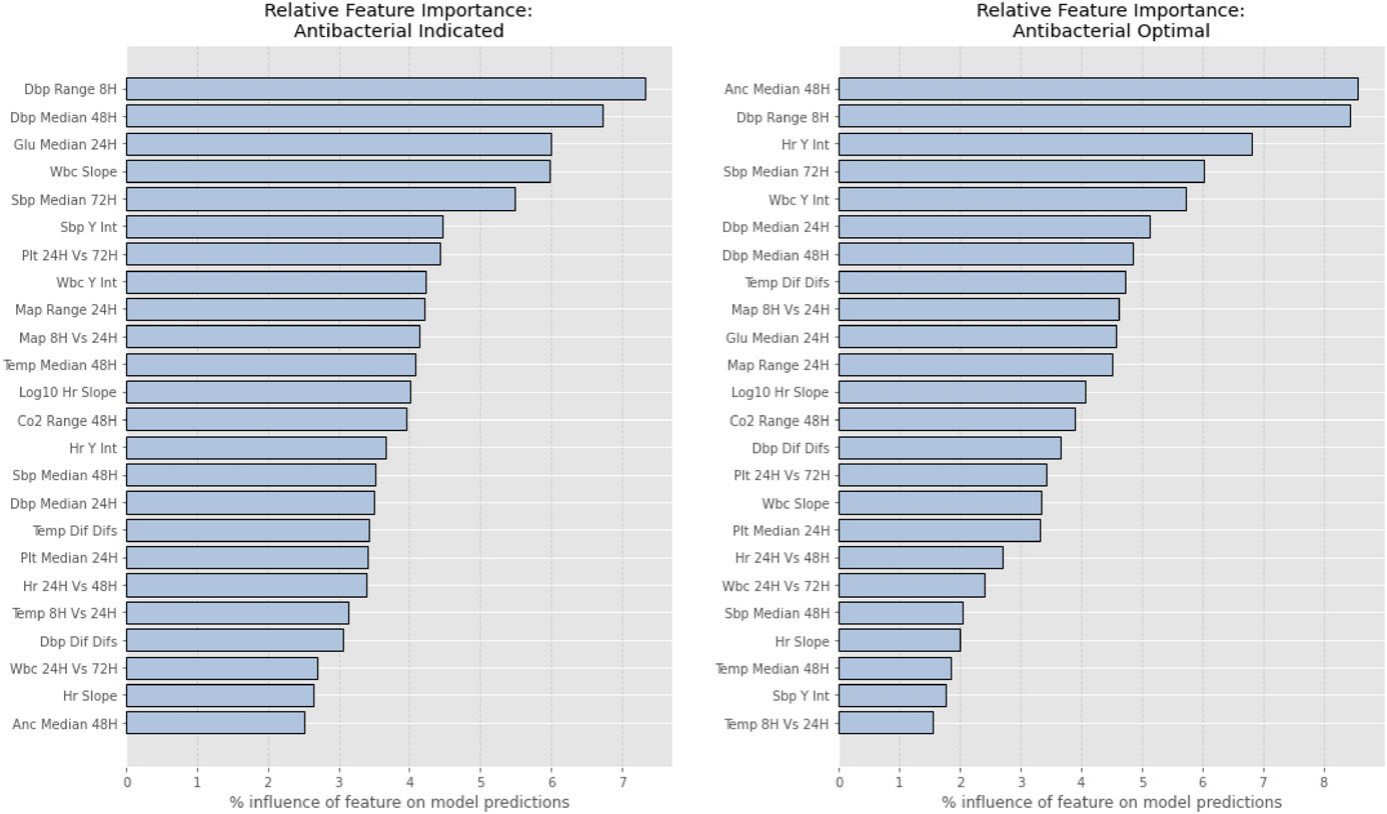
global feature performance. The x axis depicts the relative contribution of a feature as determined by SHAP values, normalized to a total of 100%.

The top 5 features identified by SHAP for each outcome were, in descending order: diastolic blood pressure range over the most recent 8 hours, median diastolic blood pressure at –48 hours, median glucose at –24 hours, estimated WBC slope, and median systolic blood pressure at –72 hours for the “indicated” outcome; and median absolute neutrophil count at –48 hours, diastolic blood pressure range over the most recent 8 hours, estimated heart rate intercept, median systolic blood pressure at –72 hours, and estimated WBC intercept for the “optimal” outcome (Figure 2).

**Figure 3.**
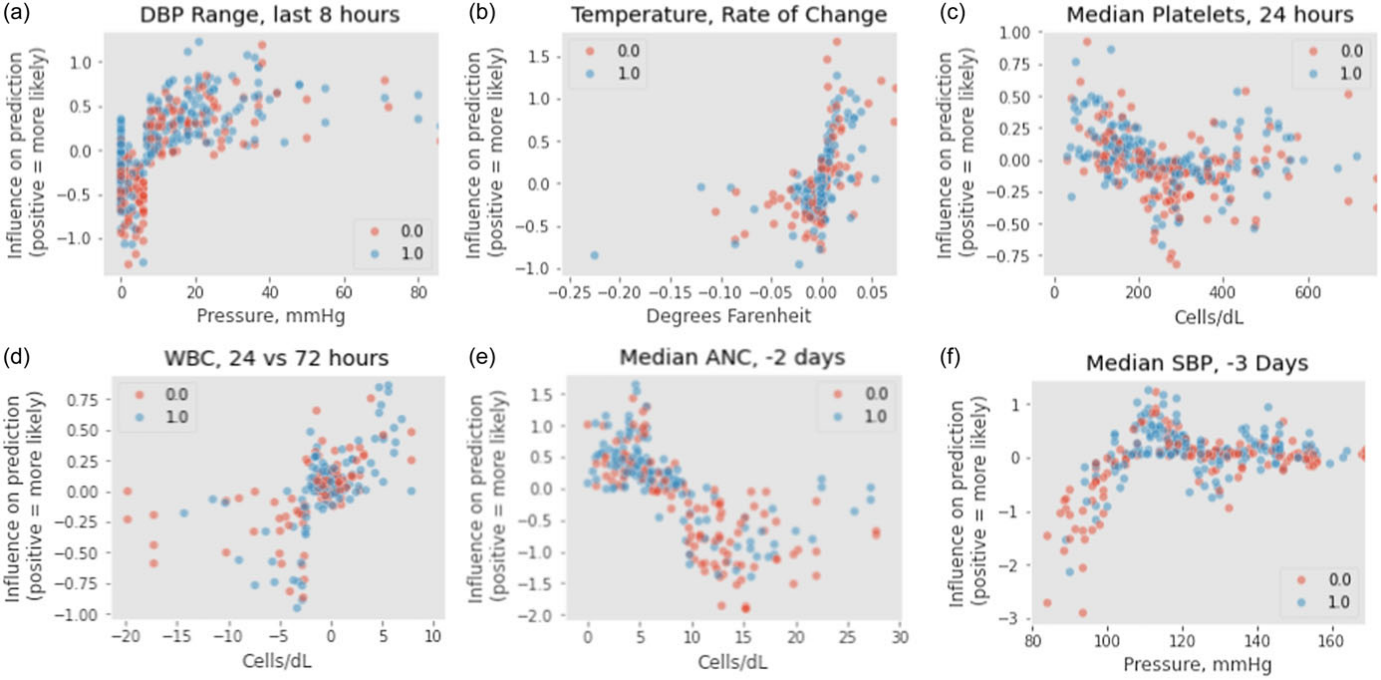
illustrative feature-prediction relationships for optimal use. Each dot represents a single prediction by the model (red dots represent orders in need of review based on ASP staff review; blue dots those which do not). The y axis represents a given value’s impact on model predictions as determined by SHAP. Negative values predict suboptimal/non-indicated orders in need for review, and vice versa.

To identify clinical correlates for those predictions, we examined the relationship between feature values (ie, clinical values) and SHAP values (ie, the values used to summarize the relative importance of those features). Illustrative results from the “optimal” model are shown in Figure 3. Figures 3a and 3b depict diastolic blood pressure range in the 8 hours prior to data collection and the rate of change in temperature. Both examples show a positive relationship between markers of clinical instability and a positive prediction (ie, a order predicted as optimal). Figure 3c shows correspondence between both elevated and lower platelet levels and positive predictions. FIgure 3d shows correspondence between a rise in white blood cell count in the 72 hours preceding review and positive predictions. Figures 3e and 3f, interestingly, show that elevated absolute neutrophil count at −48 hours and decreased systolic blood pressure at −72 hours correspond to negative predictions. A complete set of feature-variable graphs for each model is provided in the supplemental materials.

## Discussion

We describe a machine learning model that accurately stratifies inpatient antibiotic orders according to predicted likelihood of being not indicated or suboptimal and thus needing review by an ASP team. The model employs ubiquitously available data that can be automatically extracted from the EHR without the need for manual extraction, and can be interrogated to make interpretations explainable. Its predictions demonstrate accuracy over several spectra of antibiotics, although its performance appears slightly higher in community-acquired compared to hospital-acquired infections, potentially reflecting differences in complexity between infections arising in the community versus those in patients with significant healthcare exposure.

The most influential clinical features in our model include markers of hemodynamic instability, inflammation, and end-organ dysfunction. Relatively little model performance comes from antibiotic orders themselves. This emphasis on markers of infection mirrors clinicians’ approach to empiric antibiotic use; empiric treatment is life-saving in newly septic patients, and is often guided by clinical status rather than microbiological data. Interestingly, markers of instability or inflammation did not yield positive predictions more than 48 hours in advance of review. In these cases abnormal values (leukocytosis, low systolic blood pressure) actually correlate with predictions that antibiotic use might not be indicated/optimal. One potential explanation is that abnormal values in this setting can arise from preexisting non-infectious causes, such as reactive leukocytosis in malignancy or stable hypotension in a patient with cirrhosis. Alternatively, abnormal values 2–3 days prior to review may function as a proxy for prior antibiotic use, and the need to revisit prior empiric treatment.

We have previously described a machine learning approach to screen for antibiotic orders in need of ASP review, describing an approach that similarly found markers of clinical instability or systemic inflammation as key predictors.^20^ In contrast to this prior work, which used data from a single time point, our model makes use of time series data, which lends context to data that would otherwise be interpreted in isolation. This likely accounts for the increased discriminative ability of the model described in this paper.

Microbiological and MAR data contributed little to model predictions in our study. Models trained on such data alone did retain predictive ability but with poorer performance compared to vital sign or laboratory data when each was used in isolation. In contrast, work recently published by Tran-The et al, who describe a model for recommending antibiotic discontinuation, oral switch, or de-escalation, places greater emphasis on specific classes of antibiotics as well as susceptibility data.^21^ Beyond slightly different use cases for the models, this may be in part due to differences in the time between antibiotic initiation and ASP review. 60% of orders in our data set were less than 48 hours old with a median of 1.2 days, whereas data described in the authors’ paper had a mean antibiotic duration of 13.2 days in the discontinuation arm, 8.2 in their early de-escalation, and 17.3 in their late de-escalation (ibid). Microbiological data are unlikely to yield actionable information in fewer than 48 hours, a period in which decisions about antibiotics are often made empirically and influenced by markers of clinical instability or active infection. Sample size likely also influenced which features Goodman et al (2022), in a similar paper modeling antibiotic appropriateness, were able to make use of.^22^

Ultimately, the goal of a model such as this one would be to use an automated pipeline to triage antimicrobial orders in order for ASP staff to make better use of finite resources by prioritizing orders for review. Surveys of antibiotic use in United States hospitals suggest more than half of patients admitted will receive antibiotics, with a median of 776 days of therapy per 1,000 patient-days.^23^ Given the high proportion of patients receiving antibiotics, manually reviewing all orders is impractical, and our institution’s current approach is to select limited subsets of the hospital at a time in tandem with periodic point prevalence studies. While local implementation might vary in its specifics, the core data used by a model such as this one would be able to be updated on a daily basis, providing a regular, automated means for directing ASP staff’s attention to high-impact areas. This might extend ASP reach to a much broader swath of hospitalized patients.

Several limitations pertain to this model. While NAPS is a standardized and validated tool, inter-rater variability is higher at our institution for infectious syndromes with clear diagnostic and treatment guidelines compared to those where more weight is placed on clinical gestalt.^24,25^ In cases such as documented bacteremia where diagnostic criteria are straightforward, data labeling—and therefore model predictions—are likely more reliable than in less clear-cut instances like undifferentiated sepsis. Sample size, use of hospital medicine patients for training, and the single-center nature of the model, both of which reflect the time-intensive nature of ASP review, limit the model’s generalizability and complexity. Many potentially useful features, such as considering antibiotics or microbes individually rather than by spectrum of coverage, would also be more feasible with a larger sample size. While we chose to focus on clinical variables relevant to the need for antibiotics and the spectrum of coverage required, several other factors contribute to “optimal” use, including considerations around patient comorbidities, toxicities, and social factors that are not accounted for in the model. External validation, both in academic and community settings, would strengthen the case for widespread use of a model such as this one.

From an implementation standpoint, while the model was optimized to identify high-yield orders for ASP review, unstructured data such as clinical notes detailing the prehospitalization use of suppressive/prophylactic antibiotics might lead to divergent assessments. Structured data such as vital signs are key factors in identifying and diagnosing infection. However, clinical notes, radiology reports, and pathology reports lend additional context—nuances gleaned from these sources cannot be accounted for by our model. Large language models, a type of neural network that can be appropriated for text classification tasks, could be used to abstract some of this information if given a large enough corpus of training data and represent a promising development in future efforts to more effectively implement stewardship interventions, although outstanding questions remain regarding privacy and regulatory structures.^26,27^

In summary, we describe an approach toward automatically reviewing new inpatient antibiotic orders using machine learning. In contrast to labor-intensive manual review, this model uses data that can be abstracted from the medical record without the need for clinician involvement. The model’s higher throughput might be used to optimize ASP teams’ limited staffing resources; antibiotics predicted to be non-indicated or suboptimal could be prioritized, freeing up ASP resources for other tasks. Bearing our model’s strengths and limitations in mind, it is best framed as a tool for helping ASP teams triage the highest-yield orders for PAF, and defer review of those least likely to need intervention. Future extensions of this work include incorporating additional data sources, validation in broader practice settings, and investigating similar models’ impact when used to provide direct feedback to providers.

## Supporting information

10.1017/ash.2025.10142.sm001 Radakovich et al. supplementary material Radakovich et al. supplementary material

## Supplementary material

The supplementary material for this article can be found at https://doi.org/10.1017/ash.2025.10142.

## Research transparency and reproducibility

An anonymized dataset can be made available on request.
