# Supplementary material for: A machine learning model to predict optimal antibiotic use in hospital medicine patients
Source: Antimicrob Steward Healthc Epidemiol. 2025 Sep 29;5(1):e238. doi: 10.1017/ash.2025.10142 (PMC12509151; doi:10.1017/ash.2025.10142)
Supplement: Radakovich et al. supplementary material [file S2732494X25101423sup001.docx]

**Supplemental materials**

**Python packages used:**

Matplotlib 3.5.2; numpy 1.20.3; pandas 2.0.3; scikit-learn 1.0.2; scipy 1.7.3, seaborn 0.13.2; shap 0.40.0; statsmodels 0.13.2; xgboost 1.6.1

**Supplemental methods:**

Feature Engineering

Time series data such as vital signs and laboratory values pose a challenge when developing a model. Irregular collection times, high numbers of variables, and inconsistent numbers of values between samples preclude raw data from being used directly. In order to standardize model input, we handled time series data by back- and forward-filling the first and last available values, respectively, and making linear imputations between values. After imputing between values we resampled time-dependent variables to 1-hour intervals to ensure uniformity. The backfilling approach implicitly grants additional weight to the first and last observation–we considered such an approach appropriate in this context as the presenting condition and current status of a patient generally factor heavily into clinical decision-making. We aggregated time series data as maximum, minimum, median and range for fixed windows 8, 24, 48, and 72 hours prior to review (for brevity, negative values, e.g., “-72 hours” are used in the text). We selected these windows to capture relevant time spans during which new bacterial infections might become clinically apparent. To account for different durations between the time of antibiotic initiation and review, we also used a time-varied window spanning from antibiotic initiation to the end of data capture, summarizing change over time via a linear slope estimate. Biologically implausible values were removed prior to processing in order to account for measurement or transcription errors. Missing categorical values were addressed via insertion of a dummy variable at the time of model training. A schema of time series data processing is visually depicted in supplemental figure S1 (a-b).

Data labeling via the National Antimicrobial Prescribing Survey (NAPS) tool encoded antibiotic orders adjudicated as “optimal” and “adequate” by NAPS with labels of 1; “suboptimal” or “inadequate” orders were encoded with 0, and those with insufficient data were not used. The three reviewers labeled non-overlapping portions of the dataset.

Due to sample size constraints, rather than use individual antibiotics as variables we summarized spectra of coverage with the United States Centers for Disease Control-defined groups of broad-spectrum community acquired or broad-spectrum hospital acquired sepsis (BSCA and BSHA) and gram-positive coverage [(1)](https://www.zotero.org/google-docs/?64K5cY). Specific coverage for presumptive extended-spectrum beta-lactamase (ESBL) producing organisms and *Pseudomonas aeruginosa* (PsA) were also used as variables. We used the antibiotic spectrum index (ASI) described by Gerber, et al (2017) to capture the total breadth of antibiotic coverage a patient received on a given day [(2)](https://www.zotero.org/google-docs/?1IByaR).

Sample size also placed constraints on microbiological data. We aggregated culture data according to: site (blood, urine, other sterile site); whether the organism was considered an uncommon pathogen (e.g., *Staphylococcus epidermidis*) a sometimes pathogen (e.g., Viridans group streptococci) or almost certain pathogen (e.g., *Staphylococcus aureus*); isolation of methicillin-resistant *Staphylococcus aureus* (MRSA), ESBL or PsA; and finally whether the use antibiotics with activity against ESBL, PsA, or MRSA corresponded to isolation of those organisms.

We selected XGBoost, a machine learning algorithm that uses an ensemble of small decision trees to make predictions based on its superior performance in similar use cases [(4)](https://www.zotero.org/google-docs/?777Knx). We trained the model using a 10-fold cross-validation scheme, using 10 unique 90%-10% partitions of the data. In each case using the 90% portion to train the model and the 10% portion to assess its performance. Model performance was assessed via area under the receiver operating characteristic curve (AUROC), with 95% confidence intervals calculated using 1000-sample bootstrapping.

We used the SHAP package (Lundberg, 2017) to aid in model interpretation by identifying variables’ relative influence on model predictions [(8)](https://www.zotero.org/google-docs/?vOYpet). SHAP uses a game theoretical approach to interrogate machine learning models, and assesses how individual features influence model predictions, both on the dataset and individual level (ibid.). Model variables were culled using a stepwise approach, wherein the least informative variables as determined by SHAP were removed and successive models trained. Final variables were selected based on the number of variables that yielded the highest rolling median AUROC after 50 repeated stepwise selections. Separate feature selection was performed for the “necessary” and “optimal” labels.

After initial model development indicated that vital sign and laboratory data strongly influenced model output, we chose the SIRS criteria as a comparator in order to query whether the mere presence of abnormal lab/vital sign data provided predictive information. We evaluated SIRS on how many out of the four total criteria a patient met in the 24 hours preceding ASP review; AUROC was determined by setting the threshold for positivity at 0 through 4 criteria.

**Tables/figures:**

| Antibiotic | n (1324) | % |
| --- | --- | --- |
| vancomycin | 262 | 60.1 |
| ceftriaxone | 173 | 39.7 |
| piperacillin-tazobactam | 169 | 38.8 |
| cefepime | 91 | 20.9 |
| metronidazole | 85 | 19.5 |
| ertapenem | 77 | 17.7 |
| doxycycline | 74 | 17 |
| meropenem | 66 | 15.1 |
| azithromycin | 45 | 10.3 |
| ampicillin | 38 | 8.7 |
| ciprofloxacin | 31 | 7.1 |
| cefazolin | 30 | 6.9 |
| sulfamethoxazole-trimethoprim | 27 | 6.2 |
| clindamycin | 25 | 5.7 |
| levofloxacin | 23 | 5.3 |
| linezolid | 23 | 5.3 |
| amoxicillin | 21 | 4.8 |
| daptomycin | 14 | 3.2 |
| penicillin | 11 | 2.5 |
| ceftazidime | 10 | 2.3 |
| ceftolozane-tazobactam | 9 | 2.1 |
| rifampin | 7 | 1.6 |
| ceftaroline | 6 | 1.4 |
| clarithromycin | 3 | 0.7 |
| cephalexin | 2 | 0.5 |
| gentamicin | 2 | 0.5 |

**Table S1: antibiotics ordered.** Note that this includes antibiotics administered before and after the time of review, thus more distinct antibiotics are listed than the sample size used for the study


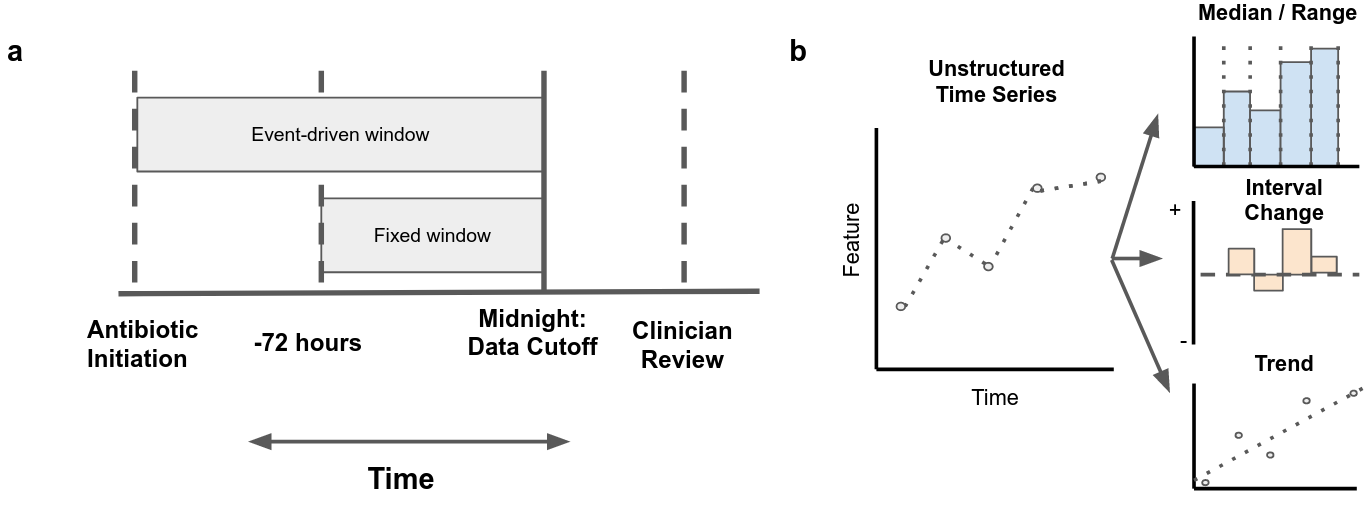


**Figure S1: schematic of approach to time series data.** A 72 hour window as well as an event-driven window were used for time series data (a), which were interpolated and summarized as median, range, interval change, and linear slope estimates (b).


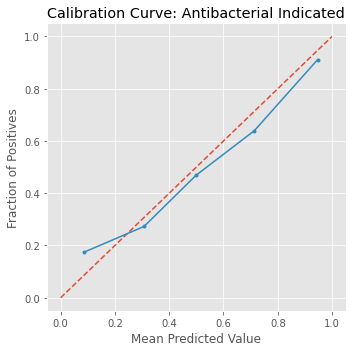


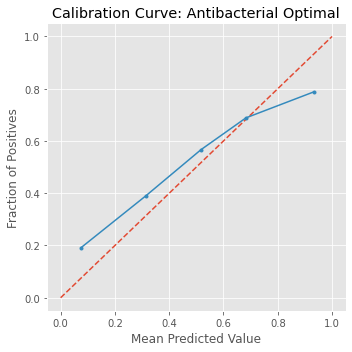


**Figure S2: calibration curves.** The points on the graph depict the proportion of the test data that are positive for a given probability predicted by the model. The dashed diagonal line represents an ideal model, e.g., one where in an infinitely large population, a 50% predicted likelihood corresponds to 50% positive rate in the population.


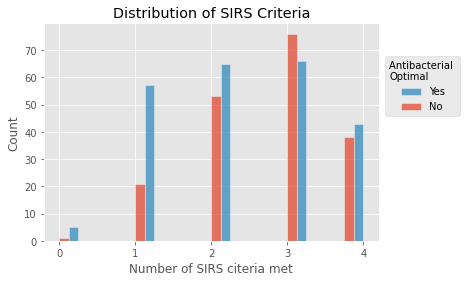


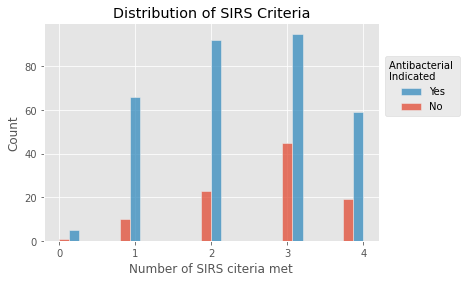


**Figure S3: SIRS criteria distribution.** Histograms depict the distribution of number of SIRS criteria met (body temperature > 38 degrees or < 36 degrees Celsius, heart rate > 90 beats per minute, respiratory rate > 20 breaths per minute, and white blood cell count > 12,000/mm^3 or < 4,000/mm^3); the two graphs are stratified by the two labels used to train the model.


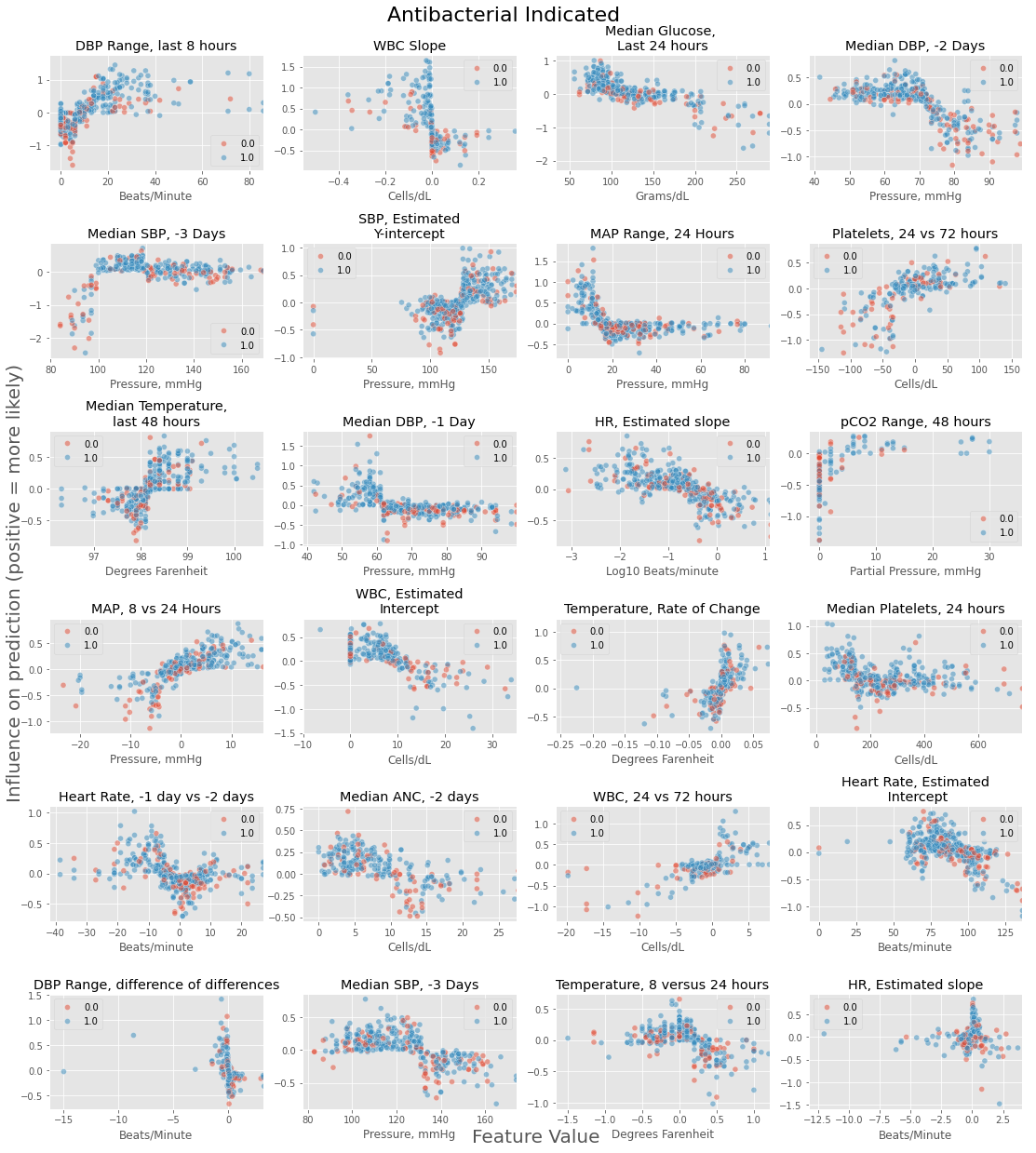


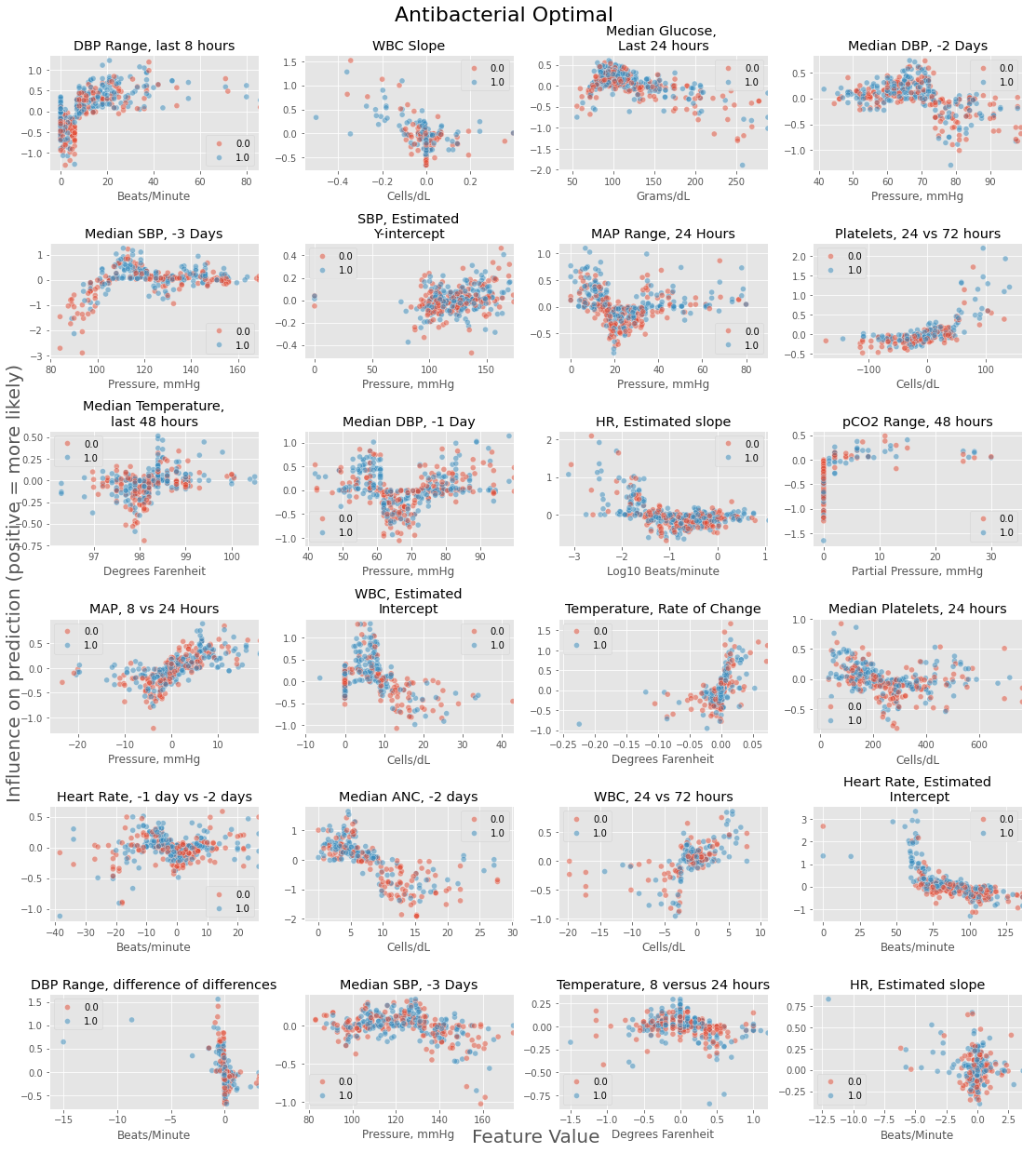


**Figue S4: complete feature-SHAP relationships**. The x-axes depict the value of a given feature, with each dot representing an individual patient. The y-axes depict the corresponding SHAP value for each patient; positive values mean that for a given patient, the feature value pushes the model toward a higher probability. Conversely, negative values push the model toward lower probabilities. Due to the nonlinear and hierarchical nature of the machine learning model, identical feature values may yield different SHAP values for different patients.

**
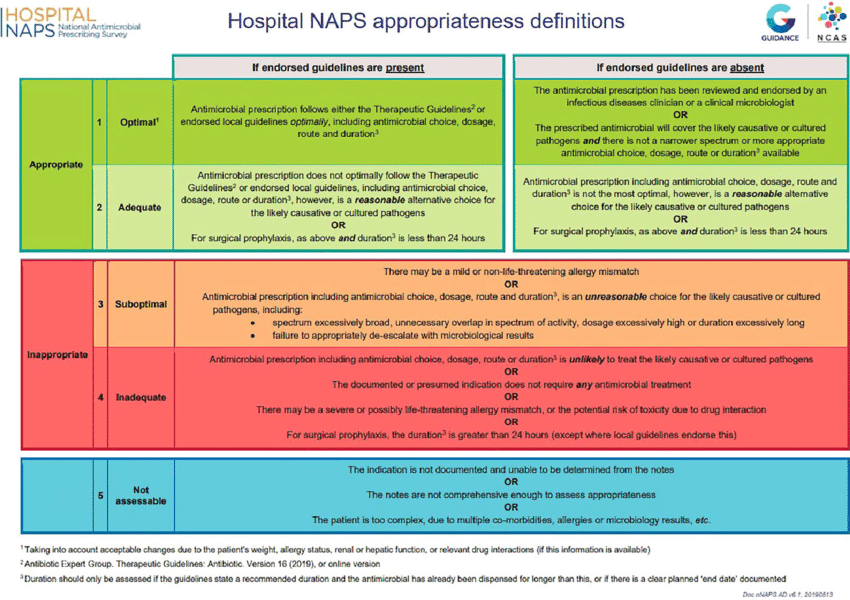
**

**Figure S5. National Antimicrobial Prescribing Survey (NAPS) tool.**

**References:**

[1. Antimicrobial Use and Resistance (AUR) Module Reports | NHSN | CDC [Internet]. 2023 [cited 2024 Jan 31]. Available from: https://www.cdc.gov/nhsn/datastat/aur-reports.html](https://www.zotero.org/google-docs/?Fkxs68)

[2. Gerber JS, Hersh AL, Kronman MP, Newland JG, Ross RK, Metjian TA. Development and Application of an Antibiotic Spectrum Index for Benchmarking Antibiotic Selection Patterns Across Hospitals. Infect Control Hosp Epidemiol. 2017 Aug;38(8):993–7.](https://www.zotero.org/google-docs/?Fkxs68)

[3. Machine learning: Trends, perspectives, and prospects | Science [Internet]. [cited 2024 Feb 8]. Available from: https://www.science.org/doi/10.1126/science.aaa8415](https://www.zotero.org/google-docs/?Fkxs68)

[4. Chen T, Guestrin C. XGBoost: A Scalable Tree Boosting System. In: Proceedings of the 22nd ACM SIGKDD International Conference on Knowledge Discovery and Data Mining [Internet]. New York, NY, USA: Association for Computing Machinery; 2016 [cited 2024 Jan 31]. p. 785–94. (KDD ’16). Available from: https://dl.acm.org/doi/10.1145/2939672.2939785](https://www.zotero.org/google-docs/?Fkxs68)

[5. Churpek MM, Snyder A, Han X, Sokol S, Pettit N, Howell MD, et al. Quick Sepsis-related Organ Failure Assessment, Systemic Inflammatory Response Syndrome, and Early Warning Scores for Detecting Clinical Deterioration in Infected Patients outside the Intensive Care Unit. Am J Respir Crit Care Med. 2017 Apr 1;195(7):906–11.](https://www.zotero.org/google-docs/?Fkxs68)

[6. Prasad PA, Fang MC, Martinez SP, Liu KD, Kangelaris KN. Identifying the Sickest During Triage: Using Point-of-Care Severity Scores to Predict Prognosis in Emergency Department Patients With Suspected Sepsis. J Hosp Med. 2021 Aug;16(8):453–61.](https://www.zotero.org/google-docs/?Fkxs68)

[7. Seok H, Jeon JH, Park DW. Antimicrobial Therapy and Antimicrobial Stewardship in Sepsis. Infect Chemother. 2020 Mar;52(1):19–30.](https://www.zotero.org/google-docs/?Fkxs68)

[8. Lundberg SM, Lee SI. A Unified Approach to Interpreting Model Predictions. In: Advances in Neural Information Processing Systems [Internet]. Curran Associates, Inc.; 2017 [cited 2024 Jan 31]. Available from: https://proceedings.neurips.cc/paper/2017/hash/8a20a8621978632d76c43dfd28b67767-Abstract.html](https://www.zotero.org/google-docs/?Fkxs68)
